# Effect of Two Methods of Remineralization and Resin Infiltration on Surface Hardness of Artificially Induced Enamel Lesions

**DOI:** 10.30476/DENTJODS.2019.77864

**Published:** 2020-03

**Authors:** Parastou Behrouzi, Haleh Heshmat, Maryam Hoorizad Ganjkar, Seyedeh Farnaz Tabatabaei, Mohammad Javad Kharazifard

**Affiliations:** 1 Dentist, Tehran, Iran; 2 Dental Material Research Center of Tehran University of Medical Sciences, Dept. of Restorative and Cosmetic Dentistry, Islamic Azad University, Tehran, Iran; 3 Dept. of Restorative and Cosmetic Dentistry, Islamic Azad University, Tehran, Iran; 4 Dept. of Restorative and Cosmetic Dentistry, Semnan University of Medical Sciences, Semnan, Iran; 5 Dental Research Center, Dental Research Institute, Tehran University of Medical Sciences, Tehran, Iran

**Keywords:** Tooth Remineralization, Resins, Hardness

## Abstract

**Statement of the Problem:**

The progression of incipient carious lesions may be simply prevented by non-invasive remineralization of lesions, eliminating the need for invasive and high-cost restorative procedures.

**Purpose:**

This study aimed to assess the effect of two commonly used remineralizing agents and resin infiltration on surface micro hardness of incipient enamel lesions at different time points.

**Materials and Method:**

In this *in vitro* study, 45 intact human maxillary central incisors were selected. After disinfection, enamel samples measuring 5x5x2.5 mm were cut out of the labial surface of the teeth. The surface of samples was polished and they were mounted in auto-polymerizing acrylic resin. According to Amaechi’s method, samples were immersed in acidified hydroxyethylcellulose system (pH= 4.5) for 96 hours to induce white spot lesions (WSLs). The baseline value of surface micro hardness of samples was measured using a Vickers hardness tester, then the samples were randomly divided into three groups (n=15) based on different remineralization methods: MI-Paste Plus, Remin Pro and ICON-Infiltrant according to the manufacturer’s instructions. All samples were stored in anti-dry mouth treatment agent during the experiment and their surface hardness was measured at 15 days (T1) and 20 weeks (T2).

**Results:**

The hardness of samples in MI-Paste Plus and Remin Pro groups significantly increased at both T1 and T2 (*p*< 0.001) but this increase was not significant in ICON group (*p*> 0.05).

**Conclusion:**

MI-Paste Plus and Remin Pro can efficiently increase the hardness of incipient enamel lesions.

## Introduction

Dental caries is a common infectious disease of the dental hard tissue, which remains a challenge in dentistry despite the widely implemented oral and dental health promotion programs [ 1 ]. Dental caries development is a dynamic process, and mineral loss occurs in the process of caries development due to an imbalance between remineralization and demineralization of tooth structure [ 2 ]. Thus, caries prevention firstly depends on the balance between these two factors [ 3 ]. The white spot lesions (WSLs) are the first clinical sign of dental demineralization. In this phase, the progression of lesion can be prevented by shifting the reactions towards remineralization. However, in case of continuation of demineralization, a dental cavity gradually forms [ 4 ]. The current concepts in dentistry emphasize on caries prevention and minimally invasive restoration of teeth [ 5 ]. Accordingly, the progression of incipient carious lesions may be simply prevented by non-invasive remineralization of lesions, eliminating the need for invasive and high-cost restorative procedures [ 6 ].

To date, many commercial products have been introduced for remineralization of WSLs such as fluoride compounds. Despite the fact that the efficacy of fluoride for remineralization of WSLs has been previously confirmed [ 7 ], more recent studies have questioned the use of fluoride for treatment of these lesions [ 8 - 9 ]. High fluoride uptake by the superficial layer of WSLs may decrease the uptake of calcium and phosphate and lead to inadequate remineralization of deeper layers [ 8 ]. Moreover, application of fluoride alone does not improve the appearance of these lesions [ 9 ]. Thus, several other compounds have been introduced for remineralization of incipient caries. Casein phosphopeptide amorphous calcium phosphate fluoride (CPP-ACPF), marketed as MI-Paste Plus, contains calcium, phosphate and fluoride stabilized by casein phosphopeptide [ 10 ]. Remin Pro is another product composed of hydroxyapatite, fluoride and xylitol [ 11 ]. ICON is the brand name of another novel product, which is believed to be minimally invasive. It replaces the lost minerals in the porous enamel with resin compounds [ 12 ]. Studies have confirmed the efficacy of the aforementioned products for remineralization of incipient enamel lesions [ 10 - 13 ].

Finding the best protocol for remineralization of incipient enamel lesions is an important step to achieve the goals of preventive dentistry with minimal invasion. Thus, this study aimed to assess and compare the effect of MI-Paste Plus, Remin Pro and ICON on surface hardness of incipient enamel lesions. The null hypothesis was that the three materials would not be significantly different in terms of their effect on surface hardness of incipient enamel lesions.

## Materials and Method

This *in vitro*, experimental study evaluated 45 human maxillary central incisors with no cracks, caries or mineralization defects, which had been extracted due to hopeless periodontal prognosis within last 3 months. The study was approved by the Ethics Committee of Islamic Azad University, Dental Faculty, Tehran (No# 191). After debridement, the teeth were immersed in 0.1% thymol solution for 48 hours and were then stored in distilled water at 37°C. Enamel samples measuring 5x5x2.5mm were cut out of the labial surface of the teeth using a cutting machine (T201A Mecatome; Presi, Germany). Samples with cracks, inappropriate size or other problems were discarded. The surface of samples was polished with 320 and 600-grit carborundum discs (Buehler, Lake Bluff, IL, USA) for 5 seconds by each disc and they were mounted in auto-polymerizing acrylic resin. The Amaechi’s method was used to induce WSLs [ 14 ]. Enamel samples were immersed in acidified hydroxyethylcellulose system (pH= 4.5) for 96 hours and were then rinsed with deionized water for 30 second and dried with gentle airflow for 5 seconds. The baseline values of surface micro hardness of samples were measured using a Vickers hardness tester (Wolpert UH930 Wilson, Aachen, Germany). The surface micro hardness was measured at three points on the surface with a distance of 100-µ from each other by applying 50kg load at a crosshead speed of 1 mm/ minute. The mean of the three values was calculated and reported as the primary Vickers hardness number (VHN). The samples were then randomly divided into three groups (n=15) for remineralization with three remineralizing agents.

In the group 1, Tooth Mousse Plus (MI Paste Plus; GC Corporation, Tokyo, Japan) was applied on the surface of samples with 1 mm thickness twice a day each time for 5 minutes. The samples were then rinsed with deionized water for 30 seconds and dried with gentle airflow for 5 seconds. This was repeated for 15 days. In the group 2, Remin Pro (VOCO GmbH, Cuxhaven, Germany) was applied as explained in the group 1.

Finally, in the group 3, ICON (DMG, Hamburg, Germany) was used according to the manufacturer’s instructions. The ICON-Etch was applied on the surface of samples for 2 minutes and was then rinsed with water for 30 seconds. The surface was dried with gentle air spray for 5 seconds. ICON-Dry was applied on the surface of samples for 30 seconds and dried with gentle air flow for 5 seconds. One layer of ICON-Infiltrant was applied on the surface of samples and remained for 3 minutes. Excess material was removed by a cotton roll and resin was light-cured for 40 seconds using a LED light-curing unit (Demetron; Kerr, USA) with a light intensity of 1600 mW/cm^2^. Next, another layer of ICON- Infiltrant was applied on the surface of samples and remained for 1 minute. Afterwards, the resin was light-cured for 40 seconds. All samples were stored in anti-dry mouth treatment agent (Kin Spray; Halitus, San Paulo, Portuguese) during the experiment that was renewed daily. The surface micro hardness of samples was measured at 15 days (T1) and 20 weeks (T2) [ 15 ]. 

Data were analyzed using repeated measures ANOVA. The changes in micro hardness after remineralization were considered as the repeated factor measure. The type of remineralizing agent was considered as the between subject variable and level of significance was set at *p*< 0.05.

## Results

**Figure1:**
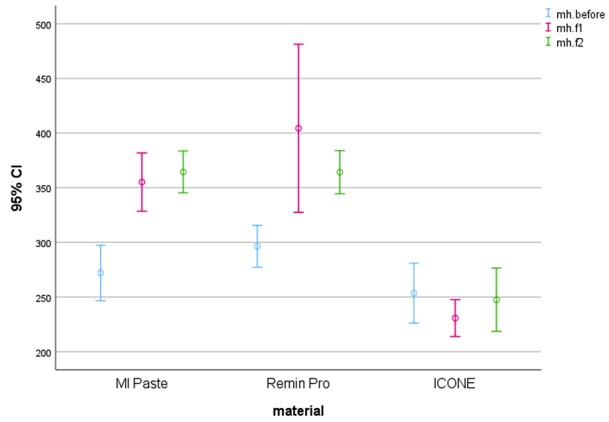
Graph representing changes in micro hardness in various groups. (Mh: micro hardness, f1: 15 days, f2: 20 weeks)

Table 1 presents the surface micro hardness of samples before and after remineralization. The results showed that MI Paste Plus significantly increased the surface micro hardness at both T1 and T2 compared to baseline (*p*< 0.001) but no significant difference was noted in this respect between T1 and T2 (*p*> 0.05). Similarly, Remin Pro significantly increased the surface micro hardness of samples at both T1 and T2 compared to baseline (*p*< 0.003) but the difference in this respect between T1 and T2 was not significant (*p*> 0.05). ICON did not increase the surface micro hardness at any time point (*p*> 0.05). Changes in micro hardness values are illustrated in Figure 1. 

Comparison of the efficacy of remineralization at T1 revealed a significant difference between three groups such that the greatest increase in micro hardness was noted following the application of Remin Pro followed by MI Paste Plus and ICON resin group (*p*< 0.001). Comparison of the efficacy of remineralization at T2 revealed a significant increase in micro hardness by MI-Paste and Remin Pro compared to ICON (*p*< 0.001). 

**Table1:** Mean micro hardness values (kg/mm2) and standard deviations (±SD) for the study groups

Micro hardness	Group 1 (MI-Paste Plus)			Group 2 (Remin-Pro)			Group 3 (ICON )			*p* value
	Mean± SD	Min	Max	Mean± SD	Min	Max	Mean± SD	Min	Max	
After Demineralization	45.84±272.04 Aa	171	327	296.42±34.64Aa	240.66	371.33	253.46±49.46Aa	169.66	306.33	>0.05
Time 1 (15 days)	355.13±48.16 Ba	236	409.66	404.35±138.96 Bb	284.33	892	230.70±30.48Ac	176.33	284.33	=0.0001
Time 2 (20 weeks)	364.44±34.59 Ba	317	424	364.20±35.52 Ba	309.66	416.33	247.49±52.31Ab	177.66	378.66	=0.0001
*p* value	<0.003			<0.001			>0.05			*p* value

Means followed by different lowercase letters show statistically significant differences between them, as compared in rows

Means followed by different capital letters show statistically significant differences between them, as compared in columns

## Discussion

This study compared the effect of two commonly used remineralizing agents and resin infiltration on surface micro hardness of incipient enamel lesions. The results showed that MI Paste Plus and Remin Pro increased the surface micro hardness of WSLs and the null hypothesis was rejected. 

Salehzadeh *et al*. [ 16 ] showed that MI Paste Plus and Remin Pro increased the enamel surface hardness, which was in agreement with our findings. 

The increasing demand for non-invasive therapeutic procedures for dental caries has encouraged researchers to assess and compare the efficacy of remineralizing agents available in the market [ 17 ]. CPP-ACP is among the commonly used remineralizing agents and its efficacy has been widely investigated [ 18 - 19 ]. MI Paste Plus is a type of CPP-ACPF available in the market that contains amorphous calcium and phosphate as well as 900-ppm fluoride, stabilized by casein phosphopeptide [ 10 ]. The phosphopeptide stabilizes the calcium and phosphate ions and creates a super-saturated state of these ions adjacent to tooth structure. Remineralization of tooth structure occurs in presence of high levels of these ions and increased pH [ 20 ]. In fact, the density of hydroxyapatite crystals increases by penetration of calcium, phosphate and fluoride ions into these crystals [ 20 ].

Leila *et al*. [ 21 ] showed MI Paste Plus to be more effective than Remin Pro in remineralization of tooth structure, which was in contrast to our findings. Although the efficacy of high concentration of calcium for dental remineralization has been previously confirmed, high concentration of calcium can cause its quick absorption by the superficial layers and thus, less remineralization occurs in deeper layers of the lesion [ 22 ]. Casein phosphopeptide present in MI Paste Plus prevents its fast deposition and stabilizes the calcium and phosphate compounds [ 20 ]. According to Vyavhare *et al*. [ 19 ] MI Paste can be used as an adjunct to fluoride but due to lower remineralization level, it cannot be used as an alternative to fluoride. It should be noted that natural saliva and dental biofilm provide a suitable environment to preserve the afore-mentioned super-saturated state of ions in the oral cavity during treatment with CPP-ACP compounds [ 23 ]. Considering the absence of these factors in vitro, the effect of these compounds can only be attributed to their application time on dental samples; this explains the difference in the results of in vivo and in vitro studies [ 24 ].

The efficacy of Remin Pro for remineralization of incipient enamel lesions has been previously confirmed25. Kamath *et al*. [ 26 ] indicated that Remin Pro increases the hardness of bleached enamel. Shetty *et al*.27 have also confirmed the efficacy of this material for increasing the micro hardness of incipient carious lesions, which is in agreement with our findings. Remin Pro is a water-based paste made of hydroxyapatite, fluoride and xylitol [ 11 ]. It seems that hydroxyapatite can fill the porosities of the incipient carious lesions. The fluoride in the composition of Remin Pro seals the tubules while xylitol exerts antibacterial effects. Thus, this compound can stop demineralization and induce remineralization of incipient enamel lesions [ 11 , 26 ].

Resin infiltration of lesions is performed aiming to fill the enamel lesion porosities and increase enamel resistance to acid attacks. This can stop caries progression and increase the strength of lesions [ 28 ]. Arslan *et al*. [ 29 ] in their study showed that infiltration of incipient enamel lesions with ICON resin increased the micro hardness and decreased bacterial accumulation, which was in contrast to our findings. Prasada *et al*. [ 30 ] in their study indicated that use of ICON resin improved the appearance of WSLs. In the ICON system, 15% HCl gel is applied to prepare the lesion surface and enhance resin infiltration into the porosities. The amount of tooth structure removed following the use of this etchant is around 40 µ; [ 30 ] while in micro-abrasion treatment of WSLs; the tooth structure is removed to 250-µ depth [ 31 ]. The ICON etchant is more effective than 37% phosphoric acid for surface erosion of lesions. Moreover, longer etching time with ICON acid compared to phosphoric acid enables more efficient resin infiltration [ 32 ]. Use of ICON-Dry (99% of which is ethanol) decreases the contact angle and viscosity and enhances resin penetration [ 32 ]. ICON-Infiltrant is a methacrylate-based resin containing BISGMA and TEGDMA. Increased concentration of TEGDMA, HEMA and ethanol increases the resin penetration coefficient [ 31 ]. In the present study, micro hardness did not increase following the use of ICON but the hardness value remained constant during the 20-week study period, which highlights the efficacy of this Infiltrant to prevent the expansion and progression of lesions. 

The prevalence of WSLs ranges from 5% to 97% in orthodontic patients. The maxillary incisors are the most commonly affected teeth [ 8 ]. Thus, the current study was conducted on maxillary central incisors. In addition, we performed Vickers micro hardness test for assessment of remineralization because evidence shows that it has high sensitivity for monitoring of enamel mineral loss [ 33 ]. Finally yet importantly, several factors such as the saliva flow, nutritional regimen, behavioral habits and oral and dental care measures can affect the speed and rate of progression of incipient carious lesions [ 34 ].

## Conclusion

Within the limitations of this *in vitro* study, it may be concluded that MI Paste Plus and Remin Pro can efficiently increase the micro hardness of incipient carious lesions. 
